# Poor-Grade Aneurysmal Subarachnoid Hemorrhage: Risk Factors Affecting Clinical Outcomes in Intracranial Aneurysm Patients in a Multi-Center Study

**DOI:** 10.3389/fneur.2019.00123

**Published:** 2019-02-27

**Authors:** Kuang Zheng, Ming Zhong, Bing Zhao, Si-Yan Chen, Xian-Xi Tan, Ze-Qun Li, Ye Xiong, Chuan-Zhi Duan

**Affiliations:** ^1^Guangdong Provincial Key Laboratory on Brain Function Repair and Regeneration, Department of Neurosurgery, The National Key Clinic Specialty, The Neurosurgery Institute of Guangdong Province, Zhujiang Hospital, Southern Medical University, Guangzhou, China; ^2^Department of Neurosurgery, First Affiliated Hospital of Wenzhou Medical University, Wenzhou, China; ^3^Department of Neurosurgery, Renji Hospital, Shanghai Jiaotong University School of Medicine, Shanghai, China; ^4^Department of Neurology, First Affiliated Hospital of Wenzhou Medical University, Wenzhou, China

**Keywords:** subarachnoid hemorrhage (SAH), intracranial aneurysm, poor-grade, risk factors, clinical outcomes

## Abstract

**Objective:** Patients with poor-grade aneurysm subarachnoid hemorrhage (SAH) have commonly been considered to have a poor prognosis. The objective of this study was to investigate the independent risk factors affecting clinical outcomes in intracranial aneurysm patients with poor-grade aneurysm subarachnoid hemorrhage (aSAH) underwent different intervention therapies.

**Methods:** A multicenter observational registry of 324 poor-grade aSAH patients treated at tertiary referral centers from October 2010 to March 2012 were enrolled in this study. The clinical data including patient characteristics on admission and during treatment course, treatment modality, aneurysm size and location, radiologic features, signs of cerebral herniation (dilated pupils), and functional neurologic outcome were collected. Clinical outcomes were assessed via a modified Rankin Scale at 12 months. Multivariate logistic regression models were used to develop prognostic models. The area under the receiver operator characteristic curves (AUC) and Hosmer-Lemeshow tests were used to assess discrimination and calibration. WAP score was developed to predict risk of poor outcome.

**Results:** Older age, female gender, ventilated breathing status, non-reactive pupil response, pupil dilation, lower GCS score, a WFNS grade of V, intraventricular hemorrhage, a higher Fisher grade, a higher modified Fisher grade, and conservative treatment were calculated to be associated with a relatively poor outcome. Multivariate analyses revealed that older age, lower Glasgow coma scale score (GCS), the absence of pupillary reactivity, higher modified Fisher grade, and conservative treatment were independent predictors of poor outcome, showed good discrimination and calibration. Patients with WFNS grade V, older age and non-reactive pupillary reactivity were predicted to have a poor outcome by WAP risk score.

**Conclusions:** A simple WAP risk score had good discrimination and calibration in the prediction of outcome. The risk score can be easily measured and may complement treatment decision-making.

## Introduction

Intracranial aneurysms are life threating vascular lesions that pose formidable treatment challenges. Of the patients who suffer from intracranial aneurysms subarachnoid hemorrhages (aSAH), nearly one third wild die in 2 weeks after hemorrhage, one third will live a dependent life and the rest will survive and be fully dependent (1).

Poor-grade aSAH (World Federation of Neurological Surgeons Grades IV and V) comprise 20–30% percent of all aSAH (2). Patients with poor-grade aSAH have commonly been considered to have a poor prognosis (3). The clinical outcome after aSAH is known to inversely correlate with admission grade, however, some reports had described a favorable outcome in a subgroup of poor-grade aSAH patients (4–6). Aggressive early intervention including urgent surgical clipping or endovascular coiling of the aneurysm may lead to favorable outcomes in a subset of poor-grade aSAH patients, but how to identify this kind of patients have conflict results.

Clinical prediction models are statistically derived tools to help clinicians predict the outcome based on highly influential indicators derived from patient history, physical examination, and laboratory and radiologic in vestigations (7). A available, high quality prediction model can help clinicians make appropriate decision, reduce the cost of care. Benefits, such as these may be particularly relevant in the management of patients with poor-grade aneurysmal subarachnoid hemorrhage since despite optimal care, mortality remains high and, among survivors, long term quality of life is disappointing.

There has been an enormous effort to work out the prognostic models predicting an outcome in patients with poor-grade aSAH over many years (8). But most models were derived by retrospective analysis of small data sets from single centers, with the outcome events too few to effectively test model assumptions and reliably select predictors for the final model. Clinical presentation of poor-grade aSAH is a highly complex process. A more comprehensive evaluation of clinical condition is essential for the proper outcome prediction.

In this study, we investigated the independent risk factors affecting clinical outcomes in intracranial aneurysm patients with poor-grade aSAH underwent different therapies. We are aimed to provide a clear description of poor-grade aSAH patient subgroup with favorable outcomes, thus help to guide for clinical treatment.

## Methods

### Study Subjects

A multicenter, prospective observational study was designed to explore the association of potential clinical risk factors with prognosis of aSAH in intracranial aneurysms patients. The study protocol was approved by the Chinese Ethics Committee of Registering Clinical Trials (ChiECRCT-2010019) and was registered with the Chinese Clinical Trail Registry (ChiCTR-TNRC-10001041). A total of 366 consecutive outpatients with intracranial aneurysms from October 2010 to March 2012 were incorporated into this study. Included patients had a previous history of poor-grade aSAH and had been treated at tertiary referral centers. Of the 366 patients, 324 patients were confirmed to have poor-grade aSAH (88.52%), and the remaining 42 patients had another grade of aSAH. The clinical data including patient characteristics on admission and during treatment course, treatment modality, aneurysm size and location, radiologic features, signs of cerebral herniation (dilated pupils), and functional neurologic outcome were collected. The inclusion and exclusion criteria were as follows:

Inclusion criteria: (1) all aSAH patients were graded (grade IV–V) according to the World Federation of Neurological Surgeons (WFNS) grading scale over a random period of time before hospitalization or at admission; (2) all patients were diagnosed with spontaneous aSAH via head CT examination or lumbar puncture after admission (the severity of aSAH was based on the head CT Fisher grade) and had a ruptured intracranial aneurysm; (3) digital subtraction angiography, computed tomographic angiography, magnetic resonance angiography or surgery confirmed the diagnosis of intracranial aneurysms, and the aneurysm led to the aSAH; (4) family members of the patients signed the informed consent and cooperated with the clinical treatment procedures, and agreed to be involved in the follow-up period; and (5) patients had complete clinical information and could be monitored during the entire experimental process with complete follow-up outcomes. Exclusion criteria: (1) WFNS aSAH grades were ator below grade III before hospitalization or at the time of admission; (2) the diagnosis of intracranial aneurysm was not clear, or the primary aneurysm was managed via surgery in the non-central unit;(3) the intracranial aneurysms was not responsible for this aSAH; (4) patients with intracranial aneurysms were accompanied by an intracerebral hematoma that was unrelated to the primary aneurysm; (5)patients exhibited severe diseases in other systems, and the estimated survival time was < 1 year, such as Carcinoma of pancreas; and (6) pregnant or lactating women were also excluded.

### Treatment Regimens

Microsurgical clipping and endovascular coiling were the main methods for the treatment of intracranial aneurysms. In addition, the operative time in this study was divided into three different groups: (1) ultra-early treatment, intracranial aneurysm rupture and hemorrhage within 24 h; (2) early treatment, 24–72 h after the rupture and hemorrhage; and (3) middle-advanced treatment, 3d after the rupture and hemorrhage. Treatment decisions for all of the included patients were predetermined by the multidisciplinary treatment team based on the consents of the family and the specific conditions of the patients. All of the treatment modalities were strictly in accordance with the patient's family's consent all of the following treatment procedures were conducted by skilled physicians with rich experience in performing operations in different hospitals.

The processing mode was divided into three categories and grouped as follows: (1) Conservative group. To be specific, patients received pure medicinal conservative treatment patients could also have received basic surgical treatment for intracranial pressure, including (1) simple external drainage surgery, which was performed in the patients with combined hydrocephalus, simple cerebral hemorrhage or hydrocephalus that affected vital signs; (2) simple hematoma removal and decompressive craniectomy, which was selected for patients with gradual increased degrees of consciousness by a focal cerebral lesion, or CT evidence of a large hematoma/an obvious shift of the midline structure (>1 cm). (2) Embolization group, including direct simple primary aneurysm embolization treatment or aneurysm embolization combined with surgical treatment to reduce intracranial pressure. This type of treatment was performed after the substantive confirmation of the presence of an intracranial aneurysm via MRA or CTA and DSA, and a consensus was reached before the operation that the patient had agreed to receive intervention therapy, general anesthesia, tracheal intubation and indwelling catheterization; in addition, such treatment might have been effective for the target patients. (3) Clipping group, including direct simple primary aneurysm clipping or aneurysm clipping combined with surgical treatment to reduce intracranial pressure and hemorrhage. Clipping surgery was adopted after the substantive confirmation of the presence of an intracranial aneurysm via MRA or CTA and DSA or in patients who had a suspected preoperative hematoma aneurysm and had agreed to receive general anesthesia, tracheal intubation and indwelling catheterization.

### Analysis of Outcomes

A dynamic follow-up evaluation was performed 12 months after the operation via telephone or letters, outpatient appointment and family visits. Clinical outcome was assessed via modified Rankin score (mRS) at 12 months by independent neurosurgeons who were not involved in the treatment of patients. Specific grading levels in mRS were as follows: (1) level 0, completely asymptomatic; (2) level 1: symptoms were present, but patients did not exhibit obvious dysfunctions and could still complete all of their daily work and live by themselves; (3) level 2: mild disability, none of the activities performed before the disease could be successfully finished by the patient alone, but the patient could still take care of their daily affairs without any additional help; (4) level 3: moderate-to severe disability and patients needed some help but could walk independently; (5) level 4, severe disability, patients could not walk independently and needed others to help with daily life activities; (6) level 5: severely disabled, bedridden, with urinary and fecal incontinence, completely dependent on others; and (7) level 6: death. In view of a high risk of morbidity and mortality, poor outcome was defined as mRS level 4 and level 5, or death. The primary indexed evaluated in the study included the functional prognosis that was measured by mRS in a follow-up period of 1 year, meanwhile, the secondary indexes were neurologic impairment, aneurysm locations, and pupillary reactivity, etc.

### Statistical Analysis

Continuous variables were presented as mean ± SD, and categorical variables as frequency (percentage). Univariate and multivariate logistic regression analyses were performed with poor outcome as the outcome variable. All clinical variables were entered into the multivariate regression model irrespective of their univariate association with poor outcome (9). The backward method was used to identify the independent predictors of poor outcome. The discriminations of prognostic models were assessed by area under the receiver operating characteristic curves (AUC). A clinical risk scoring system was developed by fitting a multivariate logistic regression model. The discrimination of the risk score was also assessed using the operating characteristic curve. An AUC of 0.7–0.8 was defined as good, 0.8–0.9 was defined as excellent, and 0.9–1.0 was defined as perfect. The calibration was assessed by the Hosmer-Lemeshow test. Data were analyzed with SPSS 22.0 (IBM Corp., Armonk, NY, USA) and SAS 9 (SAS Institute, Cary, NC).

## Results

### General Patient Information

**Figure 1:**
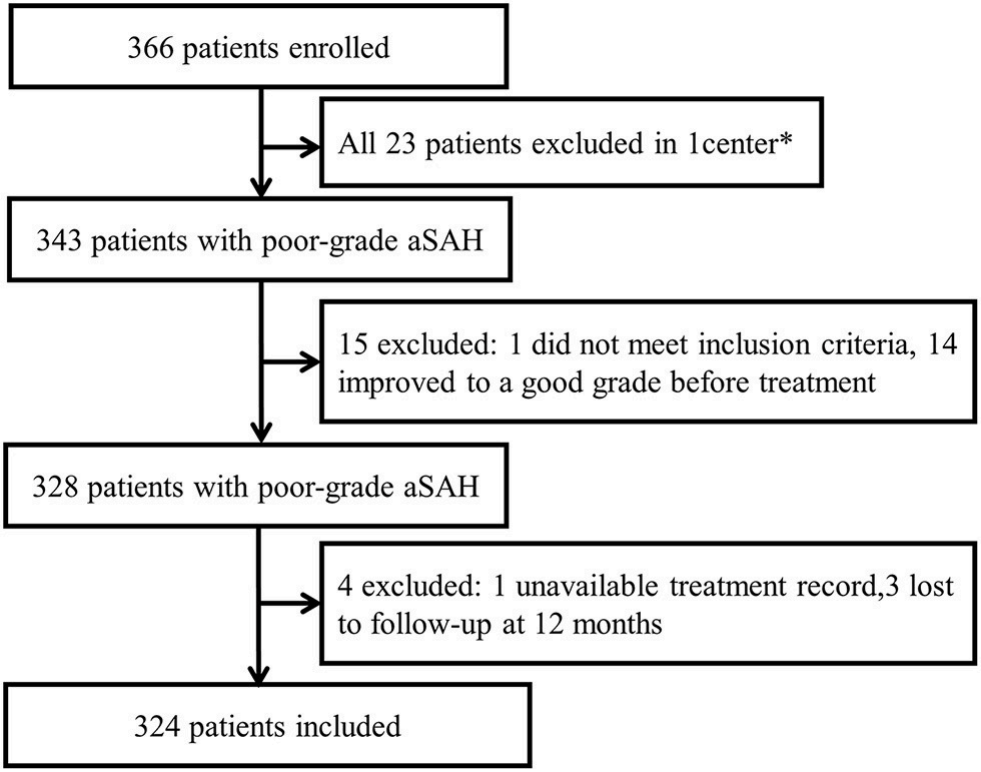
Study flow diagram. *One center was excluded because enrollment was not consecutive and patients were lost to follow-up.

The quality of data and detailed inclusion and exclusion processes were shown in Figure 1. A total of 324 patients were finally included in this study. Of the included patients, there were 159 males and 165 females, and the mean age was 55.0 ± 11.6 years. WFNS grade V was detected in 173 patients (53.4%). With respect to the treatment regimens, 130 patients underwent surgical clipping treatment, 136 patients underwent endovascular coiling treatment and 58 patients were treated with conservative treatment. During the 12 months' follow-up, a total of 190 patients (58.6%) had a poor outcome: 23 patients (7.1%) had a mRS score of 4, 13 patients (4.0%) had a mRS score of 5, and 154 patients (47.5%) died. The baseline characteristics of the 324 patients with poor grade aSAH are presented in Table 1.

**Table 1:** Baseline characteristics of eligible 324 cases of patients with poor aSAH.

**Variables**	**Good outcome (*n* = 134)**	**Poor outcome (*n* = 190)**	**OR (95% CI)**	***P***
**DEMOGRAPHIC**				
Women	58 (43.3%)	107 (56.3%)	1.7 (1.1–2.6)	0.021
Age (years)	52.6 ± 11.6	56.7 ± 11.3	1.0 (1.0–1.1)	0.002
**MEDICAL HISTORY**				
Hypertension	53 (39.6%)	89 (46.8%)	1.4 (0.9–2.2)	0.167
Current smoking	47 (35.1%)	51 (26.8%)	0.7 (0.4–1.1)	0.091
Diabetes mellitus	4 (3.0%)	14 (7.4%)	2.7 (0.9–8.3)	0.092
Coronary artery disease	14 (10.4%)	17 (8.9%)	0.8 (0.4–1.7)	0.581
Previous SAH	8 (6.0%)	6 (3.2%)	0.5 (0.2–1.5)	0.231
Previous stroke	5 (3.7%)	4 (2.1%)	0.6 (0.2–2.1)	0.392
**CLINICAL EXAMINATION**				
**Breathing status**				< 0.001
Spontaneous	125 (93.3%)	140 (73.7%)	1.0 (Referent)	
Ventilated	9 (6.7%)	50 (26.3%)	5.0 (2.3–10.5)	
**Pupillary reactivity**				< 0.001
Reactive	116 (86.6)	101 (53.2%)	1.0 (Referent)	
Bilaterally non-reactive	18 (13.4)	85 (44.7%)	5.4 (3.1–9.6)	
Others^*^	0	4 (2.1%)		
**Pupillary dilation**				< 0.001
None	115 (85.8%)	103 (54.2%)	1.0 (Referent)	
Unilateral dilation	16 (12.0%)	59 (31.1%)	4.1 (2.2–7.6)	
Bilateral dilation	3 (2.2%)	24 (12.6%)	8.9 (2.6–30.5)	
Others^*^	0	4 (2.1%)		
**NEUROLOGICAL EXAMINATION**				
Pretreatment GCS score	8.0 ± 2.6	5.6 ± 2.2	0.7 (0.6–0.8)	< 0.001
Pretreatment WFNS grade V	38 (28.4%)	135 (71.1%)	6.2 (3.9–10.1)	< 0.001
**RADIOLOGICAL FINDINGS**				
**Intraventricular hemorrhage**	43 (32.1%)	82 (43.2%)	1.6 (1.0–2.6)	0.044
**Fisher grade**	3.1 ± 0.9	3.5 ± 0.7	1.9 (1.4–2.5)	< 0.001
**Modified Fisher grade**	2.3 ± 1.0	3.1 ± 0.9	2.3 (1.8–3.0)	< 0.001
**Aneurysm locations**				0.099
Posterior circulation artery	20 (14.9%)	17 (8.9%)	1.0 (Referent)	
Anterior circulation artery	114 (85.1%)	173 (91.1%)	1.8 (0.9–3.6)	
**Multiple aneurysm**	20 (14.9%)	34 (17.9%)	1.2 (0.7–2.3)	0.481
**Aneurysm size (mm)**	5.8 ± 3.2	5.9 ± 3.7	1.0 (0.9–1.1)	0.720
**Aneurysm size > 10 mm**	8 (6.0%)	11 (5.8%)	1.0 (0.4–2.5)	0.930
**Treatment**				< 0.001
Conservative treatment	7 (5.3%)	51 (26.8%)	1.0 (Referent)	
Clipping treatment	55 (41.0%)	75 (39.5%)	0.2 (0.1–0.4)	
Coiling treatment	72 (53.7%)	64 (33.7%)	0.1 (0.1–0.3)	
**Surgical Timing**				0.302
Ultra-early treatment	70 (55.1%)	73 (52.5%)	1.0 (Referent)	
Early treatment	31 (24.4%)	29 (20.8%)	0.496 (0.7–2.3)	
Middle- advanced treatment	26 (20.4%)	37 (26.7%)	0.478 (0.4–1.5)	

*OR, odds ratio; CI, confidence interval; SAH, subarachnoid hemorrhage; GCS, Glasgow coma score; WFNS, World Federation Neurosurgical Societies*;

* *Others defined as pupillary changes associated with posterior communicating artery aneurysms*.

### Univariate Analysis for Predictors of Poor Outcome

Table 1 also described the results of the univariate analyses for the association between clinical variables and poor outcomes. Patients with poor outcome were assigned as poor outcome group, and the rest patients were assigned as good outcome group. In the 324 cases of patients, there were 190 patients had poor outcome comparing with 134 patients with good outcome. Age was found to correlate with clinical outcomes (95% CI = 1.0–1.1, *P* = 0.002), the average age of poor outcome group (56.7 ± 11.3) was larger than good outcome group (52.6 ± 11.6). The female poor aSAH patients was prone to poor outcome too (43.3%, 58/134 in good outcome group vs. 56.3%, 107/190 in poor outcome group, 95%CI = 1.1–2.6, *P* = 0.021). Patients with ventilated breathing status were found to have a poor outcome (6.7%, 9/134 in good outcome group vs. 26.3%, 50/190 in poor outcome group, 95%CI = 2.3–10.5 (*P* < 0.001). Patients with bilaterally non-reactive activity were shown to have a poor outcome (13.4%, 18/134 in good outcome group vs. 44.7%, 85/190 in poor outcome group, 95%CI = 3.1–9.6, *P* < 0.001). Patients with pupillary dilation (unilateral dilation or bilateral dilation) status were inclined to have a poor outcome, there were 19 (14.2%, 19/134) patients with unilateral dilation or bilateral dilation status in good outcome group comparing 83 (43.7%, 83/190) patients with unilateral dilation or bilateral dilation status in poor outcome group. Higher pretreatment GCS score and pretreatment WFNS graved V were correlated with poor outcome. The average pretreatment GCS score in poor outcome group was 5.6 ± 2.2 comparing 8.0 ± 2.6 in good outcome group (*P* < 0.001). There were 71.1% (135/190) patients in poor outcome group had WFNS graved V on admission comparing 28.4% (38/134) in good outcome group(*P* < 0.001). Intraventricular hemorrhage, higher Fisher grade, higher modified fisher grade and conservative treatment was associated with poor outcome. In this study, the aneurysm location, aneurysm size, surgical method, and timing of aneurysm were found not correlate with clinical outcomes. Collectively, older age, female gender, ventilated breathing status, non-reactive pupil response, pupil dilation, lower GCS score, a WFNS grade of V, intraventricular hemorrhage, a higher Fisher grade, a higher modified Fisher grade, and conservative treatment were calculated to be associated with a relatively poor outcome in those patients (all *P* < 0.05). There were trends toward poor outcomes in patients who were currently smoking (*P* = 0.091) and in those with diabetes mellitus (*P* = 0.092).

### Multivariate Logistic Regression Analysis

**Figure 2:**
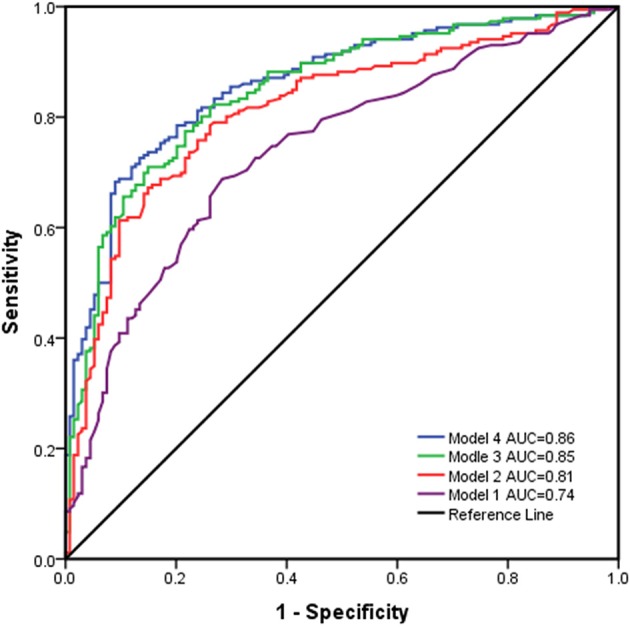
Receiver operating characteristic curves for the different prognostic models.

Multivariate regression models for the prediction of poor outcome are illustrated in Table 2. The results indicated that after controlling for confounding factors, older age, lower GCS score, the absence of pupillary reactivity, a higher modified Fisher grade, and conservative treatment were found to have significant influences on the prognostic outcomes of the patients, which indicated that they were independent predictors of poor outcomes (all *P* < 0.05). Furthermore, the prognostic models were used to predict prognostic outcomes as shown in Figure 2. The prognostic models revealed excellent discrimination with an area under curve (AUC) over 0.80, except for model 1, which included demographic data and the clinical examination (AUC = 0.74, 95% confidence interval (95%CI) = 0.69–0.79, *P* < 0.001). The discriminative ability of model 2 (AUC = 0.81, 95%CI = 0.76–0.86, *P* < 0.001) showed a relatively small trend when additional information on the modified Fisher grade (AUC = 0.85, 95%CI = 0.81–0.89, *P* < 0.001) and treatment modality (AUC = 0.86, 95%CI = 0.82–0.90, *P* < 0.001) was added. In model 2, poor outcome occurred more often in patients with lower GCS scores, older age, and an absence of pupillary reactivity (all *P* < 0.05).

**Table 2:** Multivariate analysis for predictors of poor outcome at 12 months based on the experimental design of four different models.

**Predictors**	**Model I**		**Model II**		**Model III**		**Model VI**	
	**OR (95%CI)**	***P***	**OR (95%CI)**	***P***	**OR (95%CI)**	***P***	**OR (95%CI)**	***P***
Age	1.1 (1.0–1.1)	< 0.001	1.1 (1.0–1.1)	< 0.001	1.1 (1.0–1.1)	< 0.001	1.1 (1.0–1.1)	< 0.001
**BREATHING STATUS**								
Spontaneous	1.0 (Referent)							
Ventilated	2.5 (1.0–6.1)	0.048						
**PUPIL REACTIVITY**								
Reactive	1.0 (Referent)		1.0 (Referent)		1.0 (Referent)		1.0 (Referent)	
Non-reactive	4.2 (2.1–8.5)	< 0.001	2.9 (1.4–5.8)	0.003	2.9 (1.3–6.3)	0.004	2.7 (1.2–5.9)	0.015
GCS score			0.7 (0.6–0.8)	< 0.001	0.8 (0.7–0.9)	< 0.001	0.8 (0.7–0.9)	0.001
Modified Fisher grade					2.6 (1.9–3.6)	< 0.001	2.8 (2.0–4.1)	< 0.001
Treatment modality								0.027
Conservative							1.0 (Referent)	
Surgery							0.2 (0.1–0.7)	0.007
Coiling							0.3 (0.1–0.8)	0.022

*OR, odds ratio; CI, confidence interval; GCS, Glasgow coma score*.

### Discrimination and Calibration of the WFNS Grade-Age-Pupillary Reactivity Score

Because model 2 had almost the same discrimination as models 3 and 4, an integer-based outcome risk score (WFNS grade-age-pupillary reactivity (WAP) risk score) was developed. The WAP score consists of 3 variables available after admission: WFNS grade, age (3 categories) and pupillary reactivity (Table 3). The sum of the weighted scores was used to assess the overall score and, which was ranged from 0 to 4 (WFNS grade: WFNS IV, 0; WFNS V, 1; age: < 60, 0; 60–64, 1; ≥65, 2; Pupillary reactivity: Reactive, 0; Non-reactive, 1). According to the WAP score, the predicted risk of poor outcome ranged from 25.5% for a WAP score of 0 to 96.2% for a WAP score of 4 (Table 4). The relative risk of poor outcome was 25.0 in patients with a WAP score of 4, compared to 1.0 in patients with a WAP score of 0. The WAP score showed good discrimination, with an AUC of 0.77 (95% CI 0.72–0.82, *P* < 0.001) and good calibration (*P* = 1.000). In conclusion, patients with WFNS grade V, older age and non-reactive pupillary reactivity were predicted to have a poor outcome by WAP risk score.

**Table 3:** WAP score components and weightings.

**Item**	**Score**
**WFNS GRADE**	
WFNS IV	0
WFNS V	1
**AGE (Y)**	
< 60	0
60–64	1
≥65	2
**PUPILLARY REACTIVITY**	
Non-reactive	1
Reactive	0

*WAP indicates World Federation Neurosurgical Society grade, Age, and Pupillary reactivity*.

**Table 4:** Risk of poor outcome by WAP score in the derivation cohort.

**Risk score**	**Observed risk, *N* (%)**	**Predicted risk (%, 95%CI)**	**Relative risk (95%CI)**
0	24/84 (28.6)	25.5 (18.4–34.2)	1.0 (Referent)
1	33/78 (42.3)	50.2 (43.7–56.6)	1.8 (1.0–3.5)
2	87/111 (78.4)	74.7 (68.1–80.4)	9.1 (4.7–17.4)
3	36/40 (90.0)	89.7 (83.5–93.7)	22.5 (7.2–70.1)
4	10/11 (90.9)	96.2 (92.0–98.3)	25.0 (3.0–206.1)

*CI, confidence interval*.

## Discussion

Patients with poor-grade aSAH have a poor outcome and high mortality (10). Though early intervention and aggressive treatment has improved outcome in the past years, it is controversial because most of the patients leaved hospital severely disabled (11, 12). However, some reports have shown that a subgroup of poor-grade aSAH patients might have a favorable outcome (4–6). Many risk factors have influence on the clinical outcomes of aSAH patients. Thus, this multicenter, prospective observational study was designed to explore the association of potential clinical risk factors with prognosis of aSAH in intracranial aneurysms patients. In the 324 cases of patients enrolled in this study, older age, female gender, ventilated breathing status, non-reactive pupil response, pupil dilation, lower GCS score, a WFNS grade of V, intraventricular hemorrhage, a higher Fisher grade, a higher modified Fisher grade and conservative treatment were calculated to be associated with a relatively poor outcome in aSAH patients.

Age was a strong factor correlated with clinical outcomes of aSAH patients, in this study, older patients were prone to have a poor prognosis. This was demonstrated in a number of previous studies (13–15). In the univariate analysis, female patients had poorer outcomes than male patients, probably because of the increased risk of cerebral vasospasm in female patients after aSAH (16). Intraventricular hemorrhage was also associated with poor outcome, probably because of the high risk of cerebral infarction (17). In our study, ventilated breathing status, non-reactive pupil response and pupil dilation were correlated with poor outcome in aSAH patients. Ventilated breathing status, non-reactive pupil response and pupil dilation were factors related to physical condition and severity of illness of aSAH patients. Thus, a bad physical condition and more severity of illness were prone to have a poor outcome. Pretreatment WFNS grade was used to assess aSAH because WFNS grading scale had better interobserver and intraobserver reliability than the Hunt and Hess scale. A large retrospective study showed that pretreatment WFNS grade V was correlated with poor outcome in aSAH patients (18). In our study, we had demonstrated that WFNS grade V was a good predictor for poor outcome, corresponded with previous studies (19). In the subsequent multivariate analysis, older age, a lower GCS score, an absence of pupil reactivity, a higher modified Fisher grade, and conservative treatment were independently associated with poor outcomes in patients with poor-grade aSAH. Furthermore, an integer-based outcome risk score (WFNS grade-age-pupillary reactivity(WAP) risk score) was developed to predict risk of poor outcome. Our prognostic models were derived from a large number of patients in a prospective registry, which differs from other investigations. In addition, standard neurological grades, outcome assessment scores and risk factors were systematically collected from patients at all sites. The above-mentioned risk factors were also combined into a simple risk score to predict the prognostic outcome. Our results showed that the WAP risk score had good discrimination and calibration. This risk score can be easily measured in clinical practice and may complement clinical decisions about initial treatment.

In this study, the analyses of the grade IV and V aneurysm patients showed no statistical difference in efficacy between the clipping and coiling groups. Among patients with a poor preoperative grade, some studies have documented that preoperative condition is the most important risk factor, treatment modality was not a significant prognostic factor. Although coiling is less invasive than clipping surgery, and an study on vasospasm after operation showed a significantly higher risk in the clipping group, but the ischemic infarct end point showed no statistical difference (20). The patient outcomes were mostly related to the initial subarachnoid hemorrhage and its deleterious consequences. Although the treatment decisions were not based on protocol and depended on each investigator's judgment, clipping was preferably selected for small aneurysms with a wide neck and for MCA aneurysms, and coiling was preferred for larger, ICA, and posterior circulation aneurysms.

The optimum time for poor-grade patients to undergo surgery is still debated. A nationwide study found that early surgery resulted in unfavorable outcomes (21). In 1990, the International Cooperative Study demonstrated that early surgery was neither more hazardous nor more beneficial than late surgery. Neurosurgeons have generally delayed surgery in poor-grade patients to avoid technical difficulties and surgical complications because of mass lesions and intracranial hypertension (22). Several studies demonstrate that, compared with early surgery, the incidence and severity of technical difficulties, surgical complications, or surgical morbidity is similar to patients undergoing delayed surgery (23, 24). The results of some clinical research and Meta-Analysis do not provide evidence favoring either early or delayed surgery (25). In this study, there was no statistically significant association for poor outcome incidence, among ultra-early, early and middle-advanced surgery, which were in consistent with the opinions from recent studies. It seemed rational to operate at an early stage to avoid rebleeding and predispose the anti-vasospasm therapy to best effect, afford the poor-grade patient the most reasonable opportunity for a favorable outcome.

Aneurysm size and lesion location were not associated with outcome in this study. Some previous studies have identified aneurysm location as a predictor of poorer outcome with ruptured aneurysms (26–28), but these studies only included patients who treated by surgical clipping. Some researchers analyzed patients who had undergone coiling and clipping and found no significant relationship between aneurysm location and outcomes of patients with aSAH (18, 29, 30). Some investigations demonstrated larger aneurysms correlated with higher risk of rebleeding and a higher risk of poor outcome (31, 32). This is in contrast to some studies that have not observed this relationship (33, 34). A recent prospective registry study also found no relationship between aneurysm size and outcome after 12 months of follow-up (30). Patients with large aneurysms more often have a poor clinical condition on admission but the risk of clinical and surgical complications is essentially the same as in patients with small aneurysms. And the prognostic strength of aneurysm size across previous studies were also challenged by aneurysm size threshold values applied in different studies. The inconsistent results of previous studies about the effects of aneurysm size and location may be explained by the treatment selection bias.

However, potential limitations of the present study should be taken into serious consideration. First, this risk score lacks external validation in an independent population. Second, all variables that could affect the outcome might not be captured due to the restriction of sample size and the difficulty in data collection and follow-up. Pupil changes were not tracked by using a standardized product. Modern imaging modalities, such as computed tomography perfusion and ultrasound measurement, were not recorded. Finally, the follow-up period should be prolonged to investigate the long-term outcomes of poor-grade aSAH patients. Nevertheless, we found that a simple WAP risk score had good discrimination and calibration in the prediction of outcome. The risk score can be easily measured and may complement treatment decision-making.

## Author Contributions

KZ and X-XT reviewed the literature. Z-QL and YX collected the data. KZ, BZ, and S-YC carried out the statistical analysis and drafted the manuscript. C-ZD and MZ made the overall revision of the manuscript. MZ provided the concept design.

### Conflict of Interest Statement

The authors declare that the research was conducted in the absence of any commercial or financial relationships that could be construed as a potential conflict of interest.
